# Appendicitis, with Report of Cases

**Published:** 1894-06

**Authors:** Floyd W. McRae

**Affiliations:** Atlanta, Ga.; Professor of Physiology in the Southern Medical College


ATLANTA
MEDICAL AND SURGICAL JOURNAL.

VOL. XI. JUNE, 1894. No. 4.

EDITED BY
LUTHER B. GRANDY, M.D., AND MILLER B. HUTCHINS, M.D.,
WITH THE CO-OPERATION OF
H. V. M. MILLER, M.D., LL.D., VIRGIL O. HARDON, M.D.,
FLOYD W. McRAE, M.D., ARTHUR G. HOBBS, M.D.,
AND H. R. SLACK, M.D., PH.M.

### *ORIGINAL COMMUNICATIONS.*

### APPENDICITIS, WITH REPORT OF CASES.*

BY FLOYD W. McRAE, M. D.,
ATLANTA, GA.,
Professor of Physiology in the Southern Medical College.

While much has been written and said on this subject recently, I make no apology for bringing it before you, as there are many points still unsettled. This is especially true of the question of treatment. In addition to some of the results of my rather limited personal experience, I wish to present some tentative conclusions and opinions culled from papers and discussions which have fallen under my notice within the last few months.

Dr. Nicholas Senn, in the Journal of the American Medical Association, March 24, 1894, says: "It appears to me that it would be more profitable in the future, for this department of abdominal surgery, to write less concerning individual experience, and elaborate more thoroughly, upon a pathological basis, the conditions which demand operation, viz.: the reasons for the necessity of operative

*Read before the late meeting of the Georgia Medical Association.

intervention in order to convince the mass of the profession of the correctness of the ground taken by a number of surgeons, that the appendix should invariably be removed when it is the seat of an infective lesion. There are exceptions to nearly all rules, and the surgery of the appendix vermiformis has not advanced sufficiently to enable us to lay down cast iron rules *when* and *when not* to operate. Pelvic surgery has been degraded by the modern *furor operativus,* and the same fate threatens the appendix. The conscientious surgeon must bring his work in consonance with the pathological conditions which he is expected to correct or remove."

Whatever may have been the remote cause of diminished local resistance within the appendix, pathologists are almost, if not quite, agreed that the immediate or exciting cause of appendicitis is local infection. In by far the larger proportion of cases the *Bacillus Coli Communis* seems to be the direct cause of the pathological condition. At least its almost universal presence, either alone or associated with the *staphylococci,* is generally demonstrable. In the fulminant cases, where the disease runs a rapidly fatal course, as Dr. Roswell Park suggested before the American Surgical Association last June, this bacillus seems to be the active agent in producing the local as well as the general infective peritonitis. In the localized abscesses around the appendix which pursue a rather mild course, it is very frequently absent. Here there seems to be a simple pus infection. Along this line of observation must we look for the clearing up of many mooted points which now confuse the physician and surgeon.

The diagnosis of the condition in question is not always easy, and mistakes are not infrequently made by surgeons of large experience. In children it is often confounded with intestinal obstruction and sometimes, if recourse is not had to an early exploratory incision, the real condition must remain in doubt unless cleared up by an autopsy. When, however, the attack is ushered in by pain, usually epigastric, iliac tenderness, vomiting, fever and rapid pulse, the chances are that it is appendicitis.

Richardson says: "In all cases of general peritoneal infection in which the lesion is obscure, the possibility of an appendicitis must always be borne in mind. Pain is one of the most prominent as well as one of the earliest symptoms, though varying much in degree in different cases; often general in character or epigastric at the onset of the attack, it usually becomes localized within the first forty-eight to seventy-two hours in the right iliac fossa. In exceptional cases the pain becomes localized in a remote region during the whole course of the disease.

Tenderness is of much greater diagnostic value than pain. It is generally most marked directly over the site of the appendix. Individual peculiarities must be taken into account in estimating the value of tenderness. Nausea or vomiting is an almost invariable symptom and manifests itself soon after the onset of pain. The vomiting subsides in the majority of the mild cases in a few hours, though it is apt to persist in the fulminant cases where there is early infection of the general peritoneum.

The bowels may be loose or constipated in the beginning.

Richardson expresses the firm conviction that in every case where there is a localized peritonitis a perforation, either large or small, has preceded it.

The temperature may range from a very slight elevation to one hundred and four or one hundred and five degrees. The temperature is not a safe guide as to the violence of the attack, the pulse being a rather better guide. The condition is serious if it rises to one hundred and fifteen or one hundred and twenty beats per minute.

*Treatment.*—In the fulminant cases there is no question as to the proper treatment. Nothing but an early operation will save the patient. But in the milder cases there is much diversity of opinion as to how we should proceed.

Dr. J. B. Murphy, in a paper read before the Pan-American Medical Congress and published in the Journal of the American Medical Association, reported one hundred and forty-one cases operated upon by his colleagues and himself. One hundred and eight were his own individual patients. He lays down the hard and fast rule that every case of appendicitis should be operated upon in the following extract: "When should we operate? On every case of appendicitis, or better, on every case where we have present the four cardinal symptoms:

"1. Sudden attack of pain over appendix.

"2. Always nausea, frequently vomiting.

"3. Elevation of temperature.

"4. Exaggerated local tenderness in various positions occupied by the appendix."

He not only believes that the operation should be done in every case, but that it should be done immediately.

Dr. Maurice H. Richardson, after an individual experience covering one hundred and eighty-one cases, says: "Even with this large experience I still feel in grave doubt as to the proper treatment of certain cases. Many of my colleagues with large experience have expressed misgivings as to the many questions that have arisen from time to time as new or unusual conditions have appeared. The most positive assertions in connection with the treatment of appendicitis have been made, as a rule, by those of most limited experience. In my first paper upon this subject, published in *Boston Medical and Surgical Journal* of January 19, 1888, I drew five conclusions from the five cases I had seen up to that date. To one or two of these I still adhere; the others have been long since shown to be unsound."

Dr. Wm. T. Bull states his position very clearly in the following extract from the *New York Medical Record*, March 31, 1894: "All these operations have been done because of acute attacks so frequently repeated as to lead to the belief that there was a chronic appendicitis which had no tendency to disappear spontaneously or with the aid of medical measures. These attacks seriously interfered with the occupations of the patients. In most of the cases there was palpable evidence of chronic inflammation in the shape of a tumor in the iliac fossa and a history of chronic invalidism, in greater or less degree, marked by such symptoms as irregular pain or discomfort in iliac fossa increased by exertion, irregularity in action of the bowels, flatulence, pain and distention after eating. In about the same number of patients, eighteen or twenty, I have advised against operation because the attacks were at long or irregular intervals and few in number, and the condition in the interval was apparently one of good health, without tumor and without history of intestinal irregularity or pain."

Again he says: "We need more carefully recorded cases, not only of the patients who come to operation, but those who have successive attacks relieved by medical means. There are many gaps to be filled between the surgeon who operates only when there are definite symptoms of chronic disease which have been found by operation to correspond with definite lesions of the appendix and those who resort to operation after one or two attacks, in individuals who have no symptoms, because they believe future attacks must occur in the order of nature and must endanger life.

"The position of the former may be too conservative, but I think it is up to date with the knowledge we have at the present. The attitude of the latter is unscientific, as their statements are not borne out by demonstration."

There is no question in my mind that by far the largest number of cases of appendicitis get well without operation, without falling into the hands of the surgeon, many of them unrecognized by the physician.

In the simple cases where a free purge is followed by immediate relief, and where resolution takes place readily and the restoration to health seems to be complete, I do not think an operation is indicated. I believe that in the present state of our knowledge an operation is unjustifiable. I believe that I am sustained in this position by both clinical experience and *post mortem* records.

In the fulminant cases immediate operation is the only hope for the patient. Localized abscesses should be incised and drained. In "chronic relapsing" or frequently "recurring" appendicitis, the appendix should be removed in the interval between acute attacks.

When there is a localized abscess, or where there is a general infective peritonitis, the operation should consist of simple incision and drainage with thorough toilet of peritoneum and without an attempt at removal of the appendix, unless it presents in the field of operation and can be removed without breaking up adhesions which wall off the general cavity or without unduly prolonging the operation. A secondary operation for its removal is much safer than to attempt an "ideal" operation at one sitting.

Dr. Miles T. Porter has found in investigating this subject 448

cases recorded. Incision and drainage give a mortality of eighteen (18) per cent. while removal of organ during attack gives a mortality of nineteen and seven-tenths (19.7) per cent.

Dr. Wm. T. Bull has collected statistics of 450 operations done during the period of quiescence with a mortality of only 1.77 per cent. He thinks, however, a faithful report of all cases would give a mortality of five per cent. or six per cent.

My individual experience is confined to six cases of well defined appendicitis. I have seen several other cases where there were some indications of inflammatory trouble in the right iliac fossa, but no well defined lesion. They all got well. Of the six cases of appendicitis, one—a child of two years—died before I saw him after an illness of five days. From the history of the case, and a careful examination of the body, I had no doubt as to the diagnosis. Of the other five cases, two passed through one attack, one through two attacks, and one through four attacks of well marked appendicitis in my hands. In all, except one, I advised operation. All declined it. All, except one, are now in good health. The one who has had four attacks will undoubtedly require an operation later, as there is a well defined persistent tumor in the right iliac fossa.

The sixth case I present for your inspection. I first saw her on February 24th, when she complained of slight pain in the abdomen. I prescribed a dose of salts and did not see her again until March 5th, when I was called and found her suffering with violent pain in right iliac region, nausea, subsequently vomiting. Temperature 101°, tenderness well marked over appendix, no well defined tumor. A free saline purge gave very slight relief temporarily. All the symptoms increased in severity until Wednesday morning, when immediate operation was determined upon. No tumor was discoverable at any time prior to operation. Condition at time of operation extremely unfavorable. Pulse 150, temperature 102, respiration bad. Abdomen enormously distended.

Operation March 7th. Ether. Assisted by Drs. Nicolson, Childs, Hubbard and Kennedy, of Atlanta, and Dr. Tankersly, of Ellijay. On account of extreme distention and uncertain diagnosis, I made a median incision. On separating the coils of intestines at least a quart of pus gushed out covering everything. I was unable to determine at the time of operation just what the origin of the pus was. All the viscera were so matted together that I could not tell one thing from another. I then cleansed the cavity thoroughly with hot water, sponged it out well, inserted a glass drainage tube, closed the wound with silk worm gut sutures and dressed it in the usual way (gauze cotton and flannel binder). Removed glass drainage tube and inserted rubber one on fourth day.

Recovery uninterrupted except for ischiorectal abscess from blind internal fistula which developed on thirteenth or fourteenth day.

During convalescence induration in right iliac fossa was quite well marked and confirmed my early diagnosis of appendicitis. The patient is here in excellent condition and you see the result of the operation.

